# Early prevention of carrageenan-induced peripheral/spinal inflammation suppresses microglial hyperreactivity in the trigeminal spinal subnucleus caudalis and alleviates chronic facial nociception

**DOI:** 10.1016/j.heliyon.2024.e41602

**Published:** 2025-01-07

**Authors:** Toru Yamamoto, Mitsuhiro Yoshida, Yuhei Koyama, Yatendra Mulpuri, Eiji Imado, Kana Oue, Mitsuru Doi, Yoshitaka Shimizu, Naotaka Kishimoto, Hiroshi Hanamoto, Kenji Seo

**Affiliations:** a Division of Dental Anesthesiology, Faculty of Dentistry & Graduate School of Medicine and Dental Sciences, Niigata University, Niigata, Japan; b Department of Dental Anesthesiology, Division of Oral and Maxillofacial Surgery and Oral Medicine, Hiroshima University Hospital, Hiroshima, Japan; c Department of Oral and Maxillofacial Surgery, Dokkyo Medical University School of Medicine, Tochigi, Japan; d Translational Research Center, New York University College of Dentistry, New York, USA; e Department of Dental Anesthesiology, Graduate School of Biomedical and Health Sciences, Hiroshima University, Hiroshima, Japan

**Keywords:** Carrageenan, Inflammation, Orofacial chronic pain, Microglia, Trigeminal spinal subnucleus caudalis

## Abstract

In this study, we investigated the mechanisms underlying carrageenan-induced chronic pain and the therapeutic effect of the anti-inflammatory drug meloxicam. Rats were injected with 3 % carrageenan into the masseter muscle. These rats exhibited acute and chronic hypersensitivity to mechanical stimuli for 6 weeks after injection. Pre-treatment with meloxicam prevented carrageenan-induced chronic hypersensitivity. Furthermore, minocycline and dexamethasone, but not acetaminophen, suppressed carrageenan-induced hypersensitivity in the chronic phase. Microglial reactivity in the trigeminal spinal subnucleus caudalis (Vc) was assessed by immunohistochemistry 3 days after treatment. The reactivity of microglial cells in the Vc was increased in carrageenan-treated rats compared with vehicle-injected rats. Meloxicam and dexamethasone, but not acetaminophen, prevented carrageenan-induced microglial hyperreactivity in the Vc. These results suggest that early prevention of peripheral/spinal inflammation suppresses microglial reactivity in the Vc and inhibits the development of orofacial chronic pain.

## Highlights

• Carrageenan-injected rats exhibited chronic hypersensitivity.
• Early prevention of inflammation suppressed carrageenan-induced chronic hypersensitivity.
• Early prevention of inflammation suppressed carrageenan-induced microglial activation.

## 1 Introduction

Inflammation and its associated chronic pain are major causes of discomfort and disability in humans [1]. However, the detailed mechanisms underlying the development of chronic orofacial pain are not fully understood. Inflammatory pain is caused by excessive inflammation resulting from peripheral tissue injury [2]. After tissue injury, inflammatory mediators are released from various cells, leading to inflammation, mechanical allodynia (i.e., pain due to a stimulus that does not normally elicit pain), thermal hyperalgesia (i.e., increased pain from a stimulus that normally provokes pain), and edema. Mechanical allodynia and thermal hyperalgesia result from reduced activation thresholds for primary sensory neurons or increased excitability of these neurons to a given stimulus, a phenomenon known as peripheral sensitization [3,4]. Continuous nociceptive signals at peripheral nerve endings and first-order sensory neurons are transmitted to second-order sensory neurons, leading to central sensitization, in which microglial activation plays a key role [5]. Numerous recent studies have demonstrated a major contribution of spinal microglia to the pathogenesis of chronic pain [[6], [7], [8], [9], [10], [11], [12]]. Furthermore, microglia are activated by tissue damage and inflammatory stimuli [13]. However, it's still unclear that how preventing inflammatory stimuli is effective to suppress the activation of spinal microglia and to prevent the development of chronic orofacial pain.

To address this knowledge gap, in this study, we adopted a carrageenan-induced inflammatory pain model in rats as carrageenan is commonly utilized in animal models [14], and investigated whether a non-steroidal anti-inflammatory drug (NSAID), meloxicam, suppressed carrageenan-induced microglial reactivity in the trigeminal spinal subnucleus caudalis (Vc) and the development of chronic orofacial pain.

## 2 Material and methods

### 2.1 Animals

All experimental procedures were approved by the Animal Research Committee of Niigata University (approval number: SA01111) and Hiroshima University (approval number: A15–46, A22-120, A23-100-2), and were performed in accordance with the guidelines of the National Institutes of Health on animal care and use and the International Association for the Study of Pain guidelines for the use of animals in research [15]. Male Sprague-Dawley rats (6 weeks old, 200–250 g; The Jackson Laboratory, Japan) were housed as pairs and maintained under a 12/12-h light/dark cycle at about 23 °C, with ad libitum access to food and water. Efforts were made to reduce the number of animals used and minimize their suffering in all experiments based on the international tenet for animal experimentation, 3R (Replacement, Reduction, Refinement) principle. All injections were given under sevoflurane or isoflurane anesthesia.

### 2.2 Treatment procedures

A sterile suspension of 3 % carrageenan (100 μL, diluted with sterile saline; Otsuka, Japan) was injected into the right side of the masseter muscle using a 25-gauge needle (carrageenan group). In the vehicle group, which served as the control group, the carrageenan solution was replaced with an equal volume of saline.

#### 2.2.1 Minocycline administration

Intraperitoneal (*i.p.*) administration of microglial inhibitor, minocycline hydrochloride (40 mg/kg, diluted in saline; Wako, Japan), was performed to suppress increased microglial reactivity in the subnucleus caudalis (Vc) in carrageenan-induced inflammatory pain model [16]. The concentration of minocycline was determined according to a previous study [17]. The effects of minocycline on the acute pain phase of carrageenan were evaluated by administering minocycline daily for 7 days, starting on the day of the carrageenan injection. Minocycline effects on chronic pain phase were evaluated by administering minocycline daily from 1 to 2 weeks after carrageenan injection.

#### 2.2.2 Meloxicam, dexamethasone, and acetaminophen administration

To determine if increased spinal (Vc) microglial reactivity is driven by persistent masseter inflammation induced by carrageenan, the non-steroidal anti-inflammatory drug (NSAIDs) meloxicam (2 mg/kg, *s*.*c.;* Boehringer Ingelheim, Germany) [18], the steroidal anti-inflammatory drug dexamethasone (0.3 mg/kg, *s.c.*; Nippon Zenyaku Kogyo CO.,LTD., Japan) [19], and acetaminophen, which has little anti-inflammatory effect (200 mg/kg, *i.p.*; Acelio, TERUMO, Japan) [20], were injected into the left side of the masseter muscle prior to the carrageenan injections.

### 2.3 Behavioral assessment

Rats were acclimated to the testing apparatus for 1 week before measuring head withdrawal thresholds (HWT) in the orofacial region. Baseline HWT measurements were made before carrageenan injections and 4, 8 and 24 h post injection, and then once every week (for 6 weeks) post injection. HWTs were measured using an electronic von Frey anesthesiometer (IITC 2390, IITC Life Science Inc., USA). HWTs for each rat were estimated by calculating the average of 5 trials, with each trial performed at least 1 min apart.

### 2.4 Immunohistochemical analyses

On day 3 after injection (saline, carrageenan, carrageenan + meloxicam, carrageenan + acetaminophen, carrageenan + acetaminophen + dexamethasone) treatment, the brain stem, including the Vc, was harvested. Rats were deeply anesthetized with a combination of anesthetics (2.5 mg/kg butorphanol, 0.375 mg/kg medetomidine, and 2 mg/kg midazolam, *i.p.*) and transcardially perfused with phosphate-buffered saline (PBS) and 4 % paraformaldehyde. The harvested brain stems, including the Vc (−1.0 mm to −4.0 mm caudal to the obex) were postfixed with 4 % paraformaldehyde in 0.1 M phosphate buffer for at least 48 h. After soaking in 20 % sucrose in PBS overnight for cryoprotection, the tissues were embedded in Tissue Tek OCT compound (Sakura Finetek, Japan) and frozen at −80 °C. Frozen sections were cut transversely at 30 μm thickness using a cryostat (CM1850, Leica Biosystems, Germany), followed by fluorescence staining.

Immunohistochemistry was performed on tissues containing the Vc to investigate microglial immunoreactivity using Iba1 in each treatment group. The sections were washed and blocked for 1 h with normal goat serum (5 %) diluted in PBS (0.01 M) with Triton X (0.2 %) to avoid non-specific binding of antibody to antigen on the surface of cells/tissues and to achieve higher permeability of the antibody. The sections were then incubated (24 h) with primary antibody (rabbit anti-Iba1, 1:1000; Wako, Japan). Iba1 (ionized calcium-binding adapter molecule 1) is specifically expressed in microglia in the central nervous system and is therefore used as a microglia marker [21]. Thereafter, the sections were washed and incubated (1.5 h) with goat anti-rabbit secondary antibody (Alexa fluor 594, 1:1000; Thermo Fisher Scientific, USA). After washing, sections were coverslipped with antifade reagent (VECTASHIELD mounting medium with DAPI; Vector Laboratories, USA). Images of slides were captured using a fluorescence microscope (BZ-X800, Keyence, Japan). As an antibody specificity and validation step, we confirmed no positive immunoreactivity for microglia in tissue sections incubated without the primary antibody in the preliminary experiment. Three sections from a rat containing the Vc (the section containing the highest number of microglial cells and the two immediately adjacent serial sections) were used to quantify microglial immunoreactivity using Iba1 (vehicle group, *n* = 3 rats; carrageenan (CA) group, *n* = 3 rats; carrageenan + meloxicam (CA + MELO) group, *n* = 4 rats, carrageenan + acetaminophen group (CA + ACET), *n* = 4 rats; carrageenan + acetaminophen + dexamethasone (CA + ACET + DEXA) group, *n* = 4 rats). In each section, the Vc area was analyzed using ImageJ software (NIH, USA). The number of Iba1-immunoreactive microglia in Vc was counted. The average of the three sections was used as the representative value for the individual rat.

### 2.5 Statistical analysis

Data are expressed as the mean ± SD. For behavioral experiments, two-way repeated measurement analysis of variance (ANOVA), followed by Dunnett's multiple comparison test was used to compare differences between baseline and values at each time point or between experimental groups at each time point. The area under the curve (AUC) for time–response curves for HWT were calculated for individual animals, with time on the X-axis and response on the Y-axis. Given the variability of data from animal behavior experiments, with α = 0.05 and β = 0.10, the minimal sample size was estimated as 6 using G-Power software (version 3.1, Dusseldorf, Germany). One-way ANOVA, followed by Dunnett's multiple comparison test was used to compare differences in the number of Iba1-immunopositive cells. Statistical analyses were performed using GraphPad Prism 9 (GraphPad, CA, USA). *P* < 0.05 was considered to indicate a significant difference.

## 3 Results

### 3.1 Carrageenan-induced acute& chronic hypersensitivity and the prophylactic effect of meloxicam

**Fig. 1:**
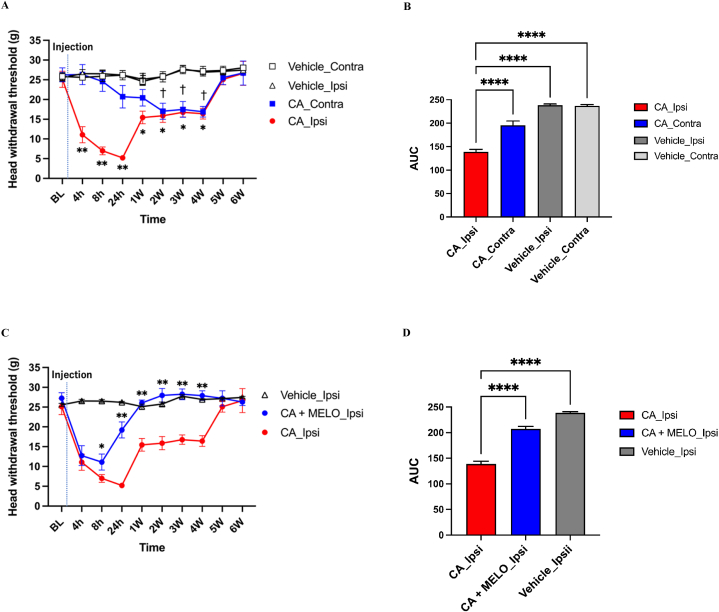
**Prophylactic effect of meloxicam against carrageenan-induced facial chronic hypersensitivity** **(A):** Change in the head withdrawal threshold (HWT) in different groups of rats. HWT of the carrageenan injection side (ipsi side) decreased significantly up to 24 h (acute phase) and persisted through 1–4 weeks after injection (chronic phase). Moreover, HWT of the contralateral side of carrageenan injection also showed a significant decrease 1 week after injection, suggesting that central sensitization and chronic hypersensitivity have occurred. Mean ± standard deviation (SD), ∗*p* < 0.05, ∗∗*p* < 0.01, vs BL in Ipsi side; ^†^*p* < 0.05, vs BL in Contra side. BL: Baseline. n = 6/group, respectively. **(B):** The area under the curve (AUC) for time–response curves for HWT. ∗∗∗∗*p* < 0.0001, vs CA_Ipsi group. **(C):** Change in the head withdrawal threshold (HWT). Note that addition of meloxicam prevented development of chronic hypersensitivity. Mean ± standard deviation (SD), ∗*p* < 0.05, ∗∗*p* < 0.01, CA_Ipsi group vs CA + MELO_Ipsi group. n = 6/group, respectively. **(D):** The area under the curve (AUC) for time–response curves for HWT. ∗∗∗∗*p* < 0.0001, vs CA_Ipsi group.

From 1 h to 4 weeks after CA injection, there were statistically (*P* < 0.05) significant differences of HWT in CA_Ipsi group compared to its BL. And interestingly, from 4 to 6 weeks after CA injection, there were also statistically (*P* < 0.05) significant differences of HWT in CA_Contra group compared to its BL. Carrageenan-injected rats exhibited hypersensitivity to von Frey hair stimulation in the acute (∼24 h) and chronic (∼1 week) phases (Fig. 1A and B).

From 8 h to 4 weeks after CA injection, there were statistically (*P* < 0.05) significant differences of HWT between CA_Ipsi group vs CA + MELO_Ipsi group. Interestingly, there was no differences of HWT between vehicle (saline) group and CA + MELO_Ipsi group, indicating the prevention of the development of the carrageenan-induced chronic hypersensitivity by meloxicam treatment (Fig. 1C and D).

### 3.2 Minocycline and dexamethasone, but not acetaminophen, suppressed carrageenan-induced chronic hypersensitivity

**Fig. 2:**
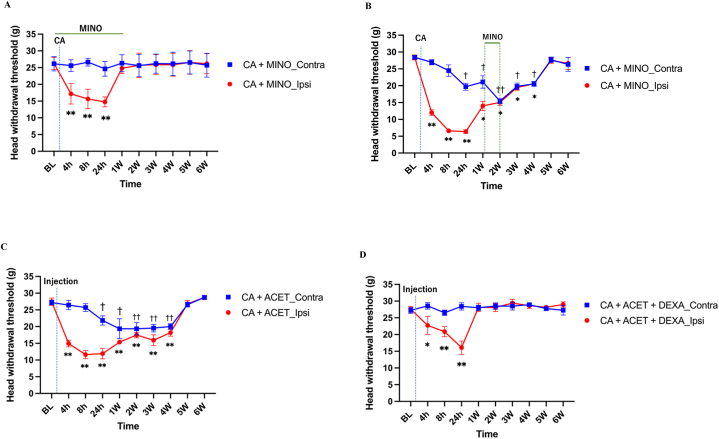
**The effect of minocycline, dexamethasone and acetaminophen on carrageenan-induced chronic hypersensitivity** Change in the head withdrawal threshold (HWT) of the Carrageenan with a microglial inhibitor, minocycline-injected side (CA + MINO_ipsi) and the contralateral side (CA + MINO_Contra). Mean ± standard deviation (SD), ∗*p* < 0.05, ∗∗*p* < 0.01, vs BL in Ipsi side; ^†^*p* < 0.05, ^††^*p* < 0.01, vs BL in Contra side. BL: Baseline. n = 6/group, respectively. Note that the administration of minocycline from the acute phase prevented the development of chronic hypersensitivity **(A)**, but the daily administration from 1 to 2 weeks after carrageenan injection in the chronic phase did not prevent the development of chronic hypersensitivity **(B)**. Moreover, administration of acetaminophen failed **(C)**, but dexamethasone **(D)**, prevented development of chronic hypersensitivity, indicating the significance of suppression of inflammation. Mean ± standard deviation (SD), ∗*p* < 0.05, ∗∗*p* < 0.01, vs BL in Ipsi side; ^†^*p* < 0.05, ^††^*p* < 0.01, vs BL in Contra side. BL: Baseline. CA; carrageenan, MINO; minocycline, ACET; acetaminophen, DEXA; dexamethasone. n = 6/group, respectively.

From 4 to 24 h after CA injection, there were statistically (*P* < 0.05) significant differences of HWT between CA + MINO_Ipsi group and CA + MINO_Contra group. But interestingly, there were no differences of HWT after 1–6 weeks, when MINO was administrated in the acute phase, indicating that suppression of microglial activation at early phase prevented the development of chronic pain. On the other hand, when MINO was administrated 1 week after CA injection (chronic phase), from 4 h to 4 weeks after CA injection, there were statistically (*P* < 0.05) significant differences of HWT between CA + MINO_Ipsi group and CA + MINO_Contra group, indicating that minocycline administration at chronic phase did not prevent the development of chronic pain. Together with the results, in this model, administration of a microglial inhibitor, minocycline starting at the acute phase, but not at the chronic phase, prevented chronic hypersensitivity (Fig. 2A and B).

Furthermore, there were statistically (*P* < 0.05) significant differences of HWT in CA + ACET_Ipsi group compared to BL at chronic phase (1 week–4 weeks after CA injection), while and there were no significant differences of HWT in CA + DEXA_Ipsi group compared to BL at chronic phase. Together with the results, dexamethasone, but not acetaminophen, prevented carrageenan-induced chronic hypersensitivity (Fig. 2C and D).

**Fig. 3:**
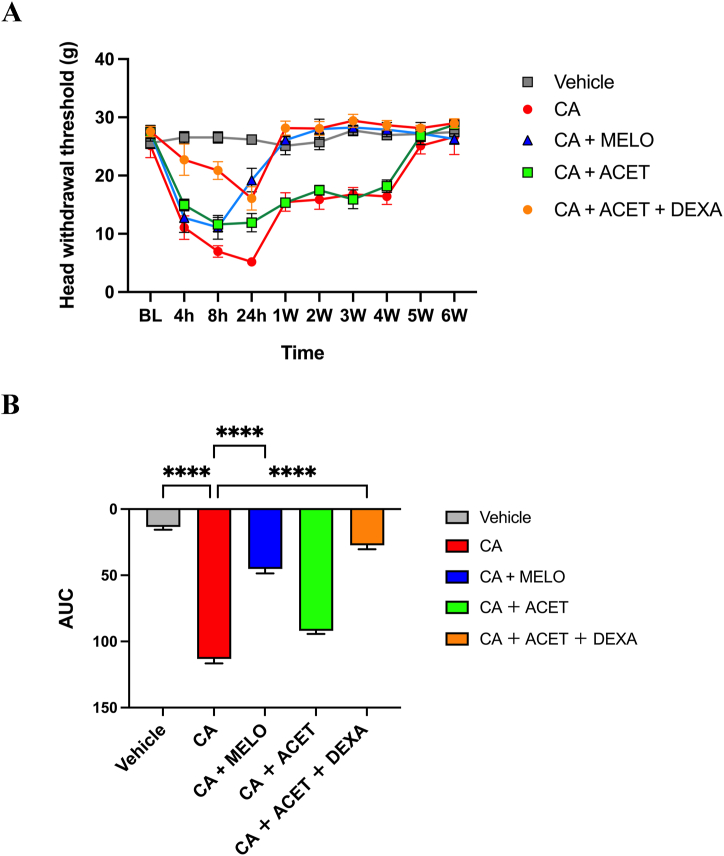
**The effect of meloxicam, acetaminophen and dexamethasone on carrageenan-induced chronic hypersensitivity** **(A):** Note that not only acetaminophen group but also meloxicam group and acetaminophen/dexamethasone group could not completely suppress carrageenan-induced hypersensitivity in acute phase, however, meloxicam and dexamethasone, which has potent anti-inflammatory effect could suppress carrageenan-induced hypersensitivity in chronic phase. Mean ± standard deviation (SD), ipsi side, n = 6/group, respectively, **(B):** ∗∗∗∗*p* < 0.001, vs CA group.

The figure summarizing the experimental groups are shown (Fig. 3A and B).

### 3.3 Meloxicam and dexamethasone, but not acetaminophen, suppresses the carrageenan-induced increase in Iba1-immunoreactive microglia in the Vc

**Fig. 4:**
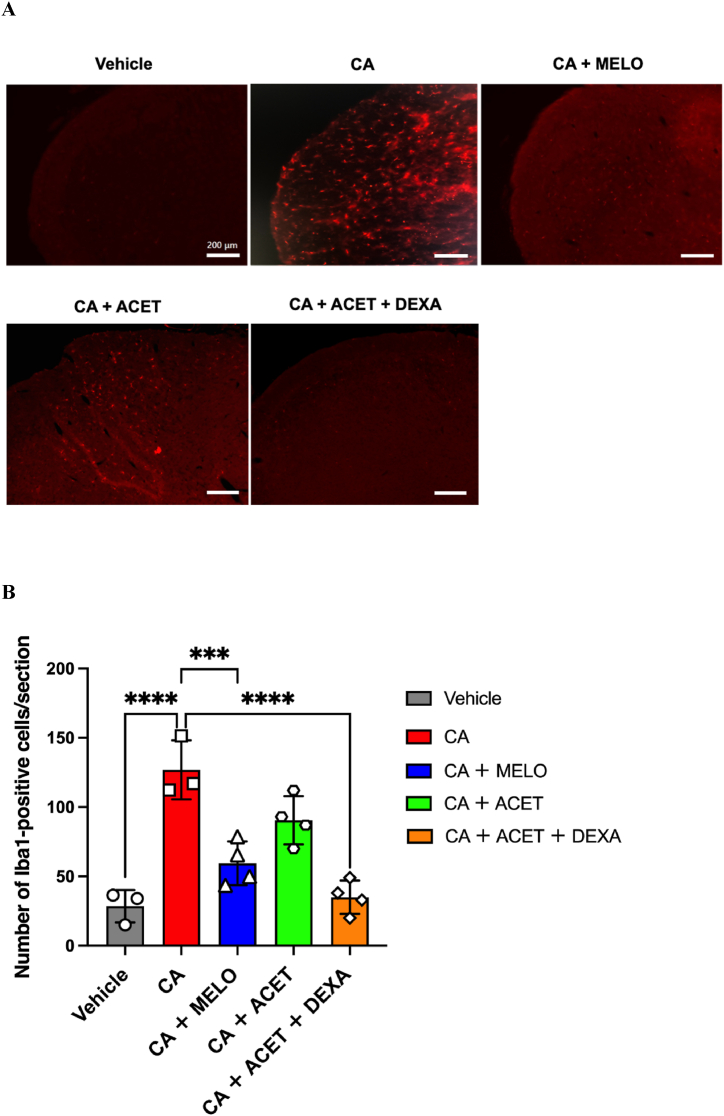
**The effect of meloxicam, acetaminophen, and dexamethasone on Iba1+ microglia in trigeminal spinal subnucleus caudalis (Vc)** **(A):** Histological images of Iba1-immunoreactive microglia in Vc among different groups of rats. **(B):** The number of Iba1-immunoreactive microglia in Vc of different groups of rats. Data are presented as mean ± standard deviation (SD). ∗∗∗*p* < 0.005, ∗∗∗∗*p* < 0.001. Note that carrageenan-induced increase in Iba1-immunoreactive microglia was significantly suppressed in carrageenan + meloxicam group, and carrageenan + acetaminophen + dexamethasone group compared to carrageenan group. CA; carrageenan, MINO; minocycline, ACET; acetaminophen, DEXA; dexamethasone. n = 3–4/group, respectively.

There were statistically (*P* < 0.05) significant differences in numbers of Iba1-positive cells between vehicle (saline) group and CA group, CA group and CA + MELO group, CA group and CA + DEXA group (Fig. 4A and B).

Carrageenan-injected rats showed a greater number of Iba1-immunoreactive cells in the Vc compared with vehicle-injected rats. Furthermore, meloxicam and dexamethasone, but not acetaminophen, suppressed the carrageenan-induced increase in Iba1-immunoreactive microglia in the Vc.

## 4 Discussion

In this study, we investigated the mechanisms involved in the development of chronic orofacial pain using the carrageenan-induced inflammatory pain model. Carrageenan is a substance found in red algae and injections of carrageenan into muscles and joints lead to both acute and chronic pain behaviors [22,23]. Our findings revealed that the threshold for detecting mechanical pressure was significantly reduced on the side where the injection was given, up to 24 h after the injection (acute phase), and this reduction persisted for 1–4 weeks after the injection (chronic phase). Furthermore, there was also a significant decrease in the threshold on the opposite side 1 week after the injection, indicating the development of central sensitization and chronic pain.

While some studies reported that injecting carrageenan into the jaw muscle of Wistar rats causes short-term but not long-lasting pain [24], other studies have shown that Lewis and Fisher inbred rats and Sprague-Dawley rats demonstrate carrageenan-induced chronic pain behavior [10,25]. The hypersensitivity to pain seems to depend on the type of carrageenan used and the injection methods [23]. There is substantial evidence to suggest that carrageenan has a long-lasting pain-inducing effect [22,26,27], which aligns with our findings. In our study, rats injected with carrageenan showed chronic hypersensitivity to mechanical nociceptive testing, and treatment with meloxicam, an NSAID, prevented the development of carrageenan-induced chronic hypersensitivity, consistent with previous studies that demonstrated the reduction of chronic hypersensitivity after spinal nerve injury accompanying with nerve inflammation [28]. Not only acetaminophen group but also meloxicam group and acetaminophen/dexamethasone group could not completely suppress carrageenan-induced hypersensitivity in acute phase, however, meloxicam and dexamethasone, which has potent anti-inflammatory effect, could suppress carrageenan-induced hypersensitivity in chronic phase. Our results suggest that the intensity and the duration of inflammation could be related to microglial activation. Regarding the experimental group, a sham injection group that would account for the potential effects of the injection procedure itself, independent of the carrageenan or drugs could help clarify the specific effects of carrageenan-induced inflammation.

Previous studies have indicated that activated microglia in a specific area of the brain stem, subnucleus caudalis (Vc), play a crucial role in the development of chronic orofacial pain [11,17,[29], [30], [31], [32], [33]]. Microglia respond to pain-inducing stimuli and inflammation in peripheral tissues as well as in a cancer pain model [16,34,35]. Therefore, in our study, we aimed to test whether reducing inflammation in the early phase could inhibit the hyperreactivity of microglia in the Vc and prevent the development of chronic orofacial pain.

It has been reported that microglia become activated in response to tissue damage and inflammation, and their activation is related to the development of chronic pain [36]. Therefore, we used minocycline, a drug that inhibits microglial activation, to examine the relationship between chronic pain behavior and spinal microglia in this model [37]. Although minocycline is typically used as an antibiotic, in our study, we used it as a microglial inhibitor [30,31,[38], [39], [40]]. The results revealed that the group in which spinal microglial activation was suppressed with minocycline did not display chronic pain behavior, indicating the involvement of spinal microglia in chronic pain behavior in this model. Additionally, our results suggest that microglia become activated during the early phase of inflammation, which is consistent with previous reports [35,41].

We also explored the effects of dexamethasone, a potent anti-inflammatory substance, and acetaminophen, which has a milder anti-inflammatory effect. The results strongly suggest that reducing inflammation is crucial for preventing the development of chronic pain behaviors [42]. Immunohistochemistry experiments showed that carrageenan increased the presence of Iba1-immunoreactive microglia in the Vc, and that the combination of meloxicam, minocycline, and dexamethasone decreased Iba1 immunoreactivity, in line with the behavioral results. The suppressive effect of minocycline on microglia has been demonstrated in the previous articles [13,37]. We wanted to investigate whether the suppression of inflammation could be the key to microglial activation or not, so we used dexamethasone, which has a strong anti-inflammatory effect, and acetaminophen, which has a weak anti-inflammatory effect, and did not include the minocycline group in the immunohistochemistry in the present study. In our preliminary study (n = 2, data not shown), we checked the activity of microglia in the chronic phase (2 weeks after carrageenan injection) and found that no robust reactivity of microglia was observed. Thus, we focused on the effect of carrageenan on microglia in the acute phase (3 days after carrageenan injection). When microglia are activated, they undergo changes in their shape and increase in number; for example, their cell bodies enlarge, and their protrusions become thicker and shorter (ramified shape to an amoeboid shape). Microglial proliferation also increases, and they undergo functional changes [[43], [44], [45], [46], [47], [48]]. In our study, we did not perform a comparison before and after activation, so we did not evaluate these morphological changes. Thus, we made statistical comparisons based on the number of cells positive for the activated microglial marker, Iba1. To investigate whether the suppression of inflammation is related to the suppression of chronic pain onset, we first used the NSAIDs meloxicam and found its correlation. As a supplementary verification, we then used ACET, which has a weak anti-inflammatory effect, but there was no statistically significant difference. We then added DEXA to ACET, which has a strong anti-inflammatory effect, and found a significant effect at least as an effect of the combination therapy, which was used as supporting data that the suppression of inflammation was the key. However, a clearer comparison would have been possible if we had observed the group that received DEXA alone.

Animal experiments showed that peripheral inflammatory stimuli cause microglial activation [34]. Although the mechanisms connecting systemic inflammatory challenge and microglial activation have not fully elucidated, several pathophysiological mechanisms have shown to play a role in this process.

First, peripheral tissue damage or inflammation leads to increased excitability of trigeminal ganglion neurons, activation of surrounding satellite glia, and neuron-glia crosstalk [49,50] resulting in neuronal hyperactivation in primary sensory nervous system [51,52].

Next, continuous noxious input from primary sensory neurons can lead to the activation of secondary neurons and microglia in the spinal Vc, which in turn can cause abnormal neuronal hyperexcitability [51]. Several pieces of evidence suggest that certain signaling pathways and molecules are involved in the activation of microglia and contribute to the development of chronic pain. Spinal inflammation induces nuclear factor-kappa B (NF-*κ*B)-p38 mitogen-activated protein kinase (MAPK) signal pathway, which exacerbates mechanical allodynia and microglial activation in carrageenan-induced inflammation [6,13,33,[53], [54], [55]]. Increased phosphorylation of p38 in microglia has been reported in a number of experimental models of inflammation pain, and inhibition of spinal p38 MAPK indeed reduced the hypersensitivity in these models [6,13]. Moreover, increased expression of brain-derived neurotrophic factor (BDNF) in microglia has been reported to increase the spine structure and synaptic connections of pain-transmitting neurons [56].

Finally, increased nociceptive information from secondary neurons in the Vc is then sent to higher centers, such as the nucleus ventralis posteromedialis and parabrachial nucleus, which modulate pain perception [8]. This flow of the process would be one of the mechanisms which underly the development of chronic pain.

Our research indicates that suppressing peripheral and spinal inflammation in the early stages can reduce microglial activation in the Vc and prevent the development of chronic orofacial pain. Once acute pain progresses to chronic pain, NSAIDs, opioids, and other medications become less effective [14,22,57]. Therefore, it is crucial to prevent the transition from acute to chronic pain [58].

Limitations of our study are that 1) this study does not account for the role of other glial cells, such as astrocytes, which are also known to play significant roles in neuroinflammation and chronic pain [17,31,59], and that 2) Drug Dose and Route Variability; this study uses fixed doses of meloxicam, minocycline, and dexamethasone thus, the potential variability in drug effects due to dosage and route should remain, and that 3) Lack of Long-Term Follow-Up; this study followed the behavioral alternation up to 6 weeks after carrageenan injections, however, more long-term observation may be needed to assess long-term outcomes in the trigeminal nerve region. It has been reported that carrageenan injection into the knee joint or muscle (spinal nerve region) induced an acute phase of inflammation followed by a chronic phase lasting up to 8 weeks was observed in SD rats, the same strain used in our present study [22].

Although the present study has translational limitations of the rodent model, including differences in physiology and potential human-specific factors, the challenge for pain researchers using preclinical models is to adequately model the complexity of the patient's pain experience in animals to elucidate and advance our understanding of the underlying mechanisms. Animal studies have the advantage of systematically examining factors associated with acute pain and with the development and maintenance of chronic pain to establish causal relationships that are often not possible in humans. Rodents are used primarily to demonstrate their sophisticated nervous systems and genetic similarities to humans [60]. Further research of the intracellular mechanisms responsible for the development of chronic pain caused by carrageenan-induced inflammation should expand our understanding.

## CRediT authorship contribution statement

**Toru Yamamoto:** Writing – original draft, Visualization, Software, Methodology, Investigation, Funding acquisition, Formal analysis, Data curation, Conceptualization. **Mitsuhiro Yoshida:** Writing – original draft, Methodology, Investigation, Funding acquisition, Data curation, Conceptualization. **Yuhei Koyama:** Methodology. **Yatendra Mulpuri:** Writing – review & editing, Conceptualization. **Eiji Imado:** Methodology. **Kana Oue:** Resources. **Mitsuru Doi:** Resources. **Yoshitaka Shimizu:** Resources. **Naotaka Kishimoto:** Writing – review & editing, Funding acquisition. **Hiroshi Hanamoto:** Resources. **Kenji Seo:** Writing – review & editing, Validation, Funding acquisition.

## Ethics statement

All experimental procedures were approved by the Animal Research Committee of Niigata University (approval number: SA01111) and Hiroshima University (approval number: A15–46, A22-120, A23-100-2).

## Data availability statement

Data will be made available on request.

## Additional information

No additional information is available for this paper.

## Funding statement

This work was supported by JSPS KAKENHI [grant numbers: 20K10116, 23K09353] to Mitsuhiro Yoshida and [grant numbers: 23K09350] to Toru Yamamoto, [grant numbers: 22K19615] to Kenji Seo.

## Declaration of competing interest

The authors declare that they have no known competing financial interests or personal relationships that could have appeared to influence the work reported in this paper.
